# MAGE-A as a novel approach in the diagnostic accuracy of oral squamous cell cancer: a case report

**DOI:** 10.1186/1758-3284-1-39

**Published:** 2009-12-16

**Authors:** Philipp Metzler, Nur Mollaoglu, Stephan Schwarz, Friedrich W Neukam, Emeka Nkenke, Jutta Ries

**Affiliations:** 1 Department of Craniomaxillofacial and Oral Surgery, University of Zurich, Frauenklinikstrasse 24, 8091 Zurich, Switzerland; 2 Department of Oral and Maxillofacial Surgery, University of Erlangen-Nuremberg, Glueckstrasse 11, 91054, Erlangen, Germany; 3 Department of Oral and Maxillofacial Surgery, University of Gazi, School of Dentistry, Emek 8.cadde, 82.sok. No.4, 06510, Ankara, Turkey; 4 Institute of Pathology, University of Erlangen-Nuremberg, Krankenhausstr, 12, 91054 Erlangen, Germany

## Abstract

**Background:**

The aim of this case report is to introduce the combined use of brush biopsy and measurement of MAGE-A expression in the diagnosis of oral squamous cell carcinoma (OSCC).

**Case report:**

We report of a 49-year old male patient who was referred to our department with a persistent-suspicious looking leukoplakia. Brush biopsy and an incisional biopsy were performed following clinical diagnosis. Histopathological examination revealed no malignancy. Expression analysis of melanoma-associated antigens A (MAGE-A) using real time RT-PCR was applied to brush biopsy materials because of the high prevalence of MAGE-A determined previously in OSCC's. Results indicated significant MAGE-A3 and A4 expression pattern. Therefore, the lesion was excised completely and an early invasive carcinoma was identified.

**Conclusion:**

These results emphasize the role of brush biopsy using a tumor marker with a high expression frequency combined with a high sensitive and high specific detection system in the early diagnosis of OSCC, particularly in widespread leukoplakias.

## Introduction

Early detection of oral squamous cell carcinoma (OSCC) at an early stage improves the five-year patient survival rate to approximately 80%. Therefore, early diagnosis is the most effective approach for reducing morbidity and mortality. In routine follow-up, the oncologists and the head and neck surgeons are often confronted with suspicious and even non-suspicious lesions of the oral mucosa, especially in pitted areas after tumor resection or post-radiation within a long observation period. Over the past decades, adjunctive methods have emerged to facilitate the diagnosis of premalignant and malignant oral lesions [1]. Until now, incisional biopsy and histopathological evaluation have been the most accurate and reliable methods for diagnosing suspicious oral lesions [2]. Histopathological diagnosis and the following surgical and/or radiochemotherapeutical approach depend on subjective recognition, identification of the most appropriate site of the ambiguous oral lesion and sufficient sampling. Therefore, incisional biopsy may have some limitations, especially in widespread lesions or areas with no clinical evidence of histopathological changes or molecular alterations (e.g. field cancerization) [3-5].

The current literature shows several adjunctive sampling and diagnostic techniques for gaining knowledge about the malignant differentiation and transformation of the oral epithelium [1,6]. In the past decade, brush biopsy of oral mucosal lesions has aroused new interest as a non-invasive cell sampling method and various new techniques for cytological analysis have been developed. Despite the wide use of this technique, conventional exfoliative cytology method displayed a limited sensitivity of between 79% to 97% and specificity from 95.1% to 99.5% [7]. Although promising analytical efforts to minimize false positive and negative results using this method have previously been made, it still has some drawbacks [7,8]. However, until now the early detection of malignant transformation of the oral mucosal lesions, especially in apparently innocuous lesions, has remained a problem [9]. Therefore, this case report aimed to advance the methodical diagnosis of exfoliative cytology in oral cancer using an objective high prevalent tumor marker in combination with a high sensitive and specific molecular detection analysis. Therefore, the present case report sought to introduce the combined use of brush biopsy and measurement of MAGE-A expression for the diagnosis of OSCC.

## Case report

**Figure 1:**
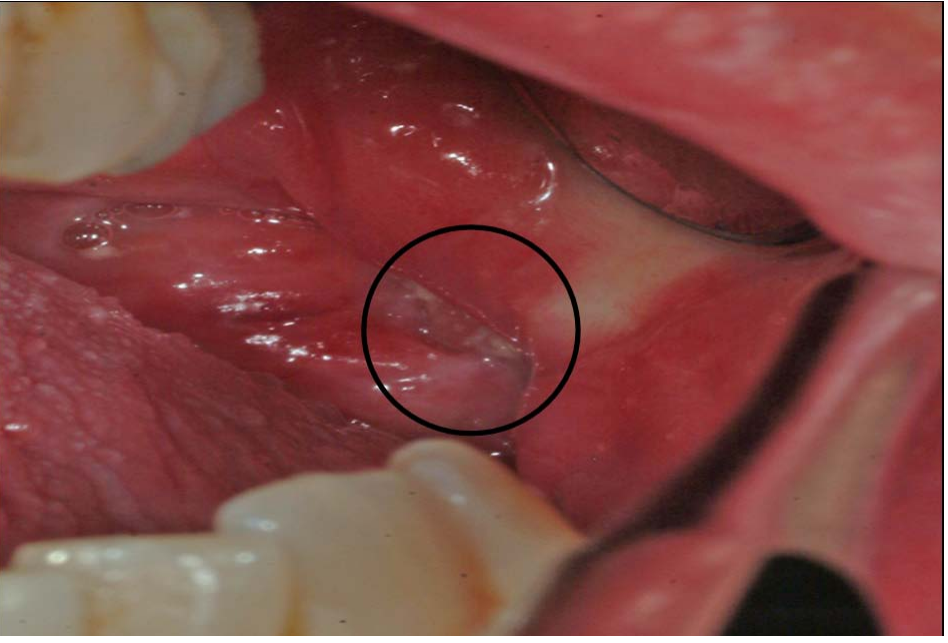
**Leukoplakia at the left palatoglossal arch**.

**Figure 2:**
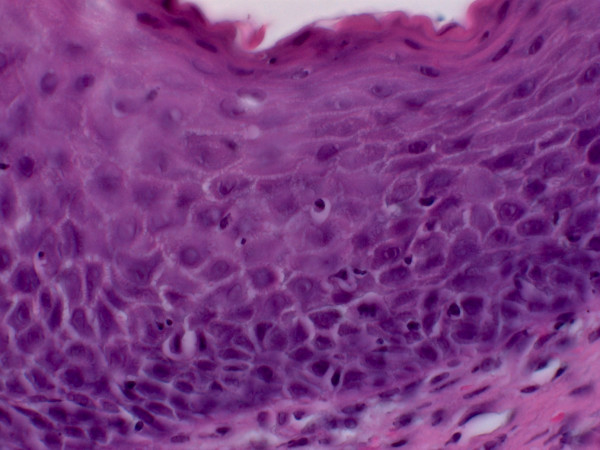
**Hyper- and parakeratosis (H&E, oil immersion, 630-fold magnification)**.

**Figure 3:**
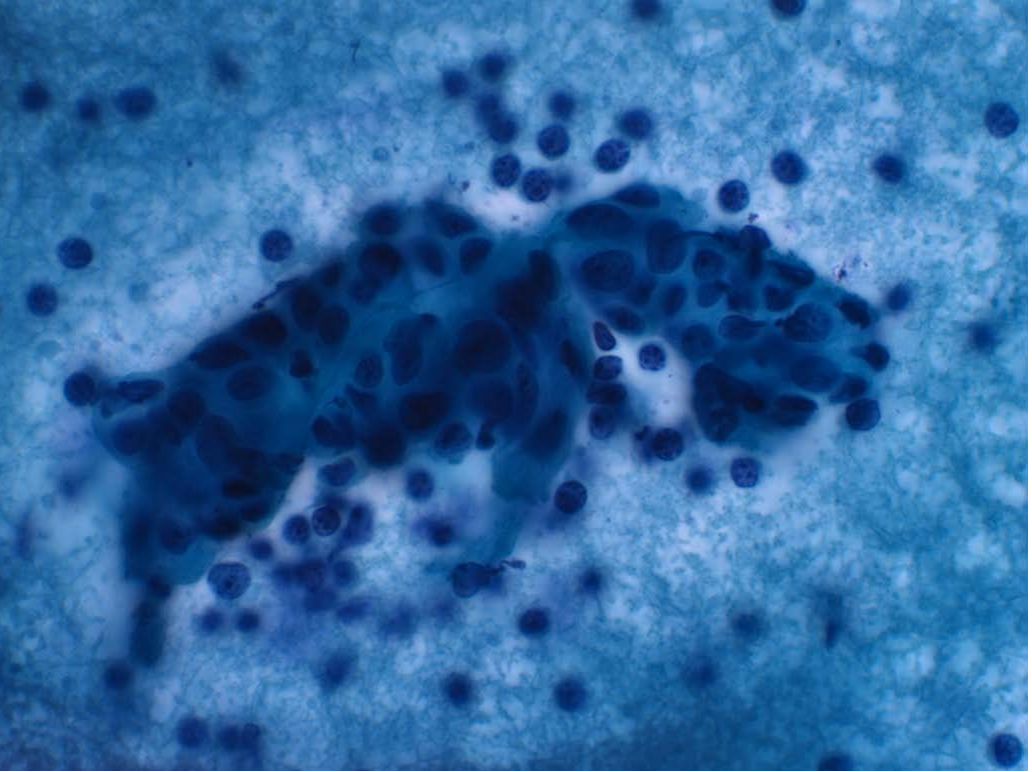
**Atypical epithelial cells with polygonal epithelial cells showing clear cytoplasm and isomorphic, oval, small nuclei (Papanicolaou stain, oil immersion, 630-fold magnification)**.

**Figure 4:**
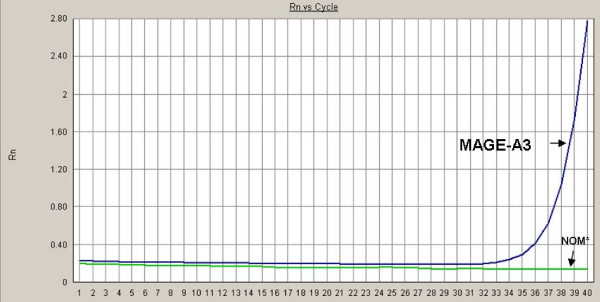
Expression analysis of MAGE-A3 and -A4 by real time RT-PCR, NOM* (Normal oral mucosa); Rn represents the raw flurescence signal over time.

**Figure 5:**
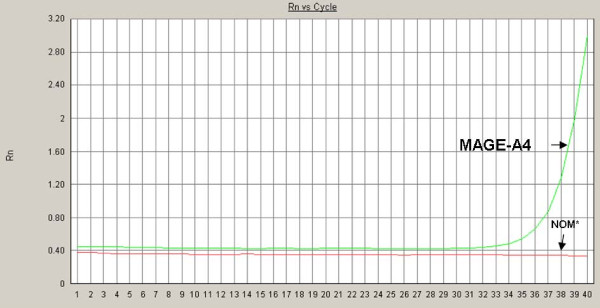
Expression analysis of MAGE-A3 and -A4 by real time RT-PCR, NOM* (Normal oral mucosa); Rn represents the raw flurescence signal over time.

**Figure 6:**
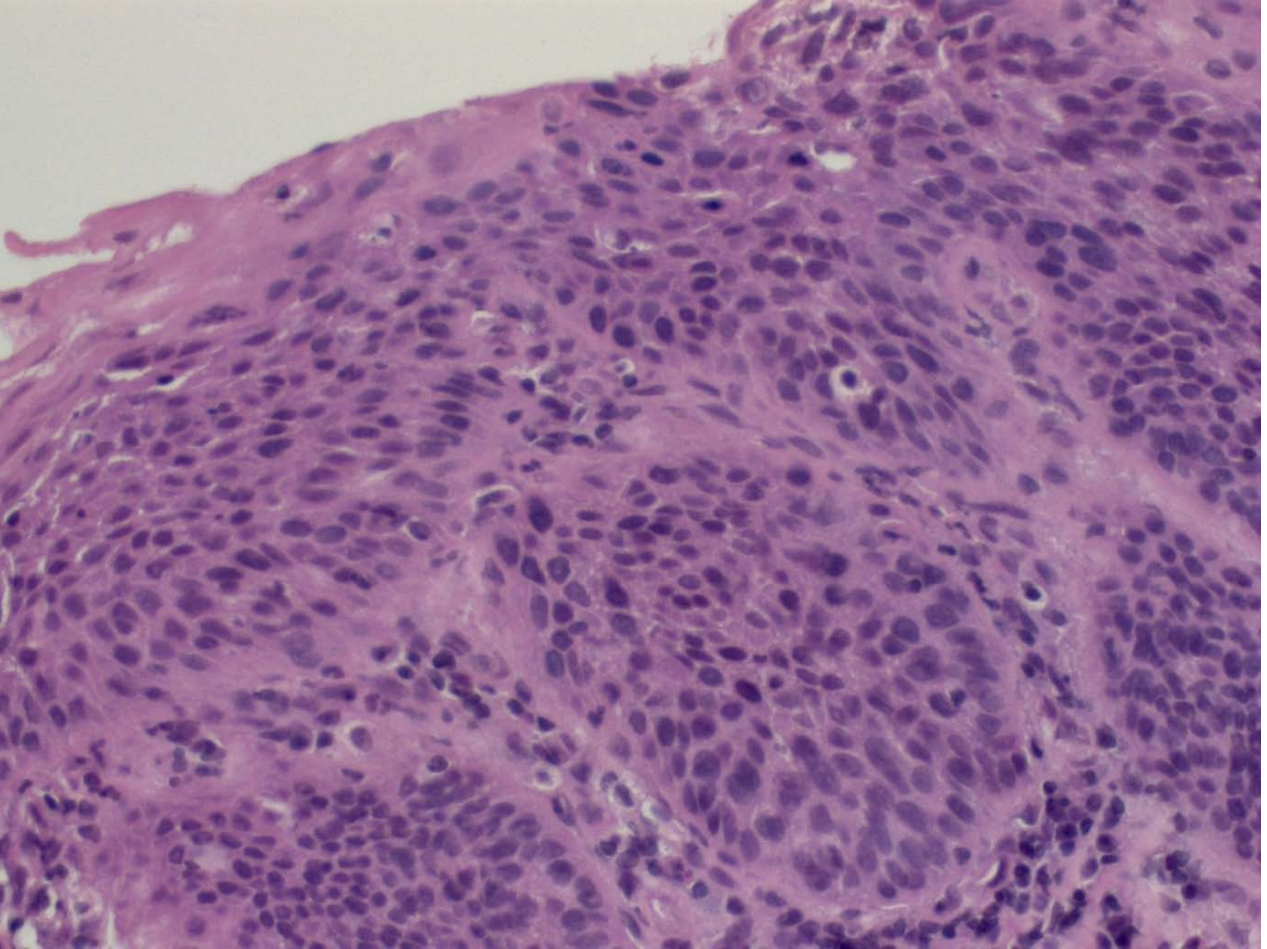
**Early carcinoma (H&E, oil immersion, 400-fold magnification)**.

A 49-year old patient was referred to the Department of Oral and Maxillofacial Surgery at the University of Erlangen-Nuremberg following OSCC of the left floor of the mouth, including the partial resection of the mandible, neck dissection and local reconstruction. The histopathological examination displayed pT1 pN0 cM0 G2 R0 status. Adjuvant radiotherapy was not carried out because of the low invasion depth of 3 mm. Patient was closely monitored during regular follow-up intervals. Five months later, leukoplakia at the patient's left palatoglossal arch was diagnosed (Figure 1). No induration was palpable. Subsequently, the lesion was biopsied by scalpel at the most suspicious site. The histological examination of the incisional biopsy showed a hyper- and parakeratosis (thickening and keratinization characterized by the retention of nuclei of the stratum corneum), but no malignancy (Figure 2). Additionally, this lesion was routinely smeared using a commercially available cytobrush (Cytobrush^® ^Plus GT, Medscand Medical AB, Sweden). Sampling was made with a moderate pressure and permanent rotation within the whole lesion in order to harvest cells. The brush was then rolled out on a slide by taking care of cell folding, air-dried and stained according to Papanicolaou. The following histopathological analysis of the specimen showed an epithelial dysplasia (atypical epithelial cells with polygonal epithelial cells showing clear cytoplasm and isomorphic, oval, small nuclei) (Figure 3). For further accuracy of methodical diagnosis of exfoliative cytology, we established a highly sensitive multimarker real time RT-PCR assay to detect the OSCC-related MAGE-A genes using a brush biopsy. As a negative control, healthy contralateral oral mucosa was smeared. The following molecular analysis revealed a significant increase of MAGE-A3 and A4 expression in the leukoplakia in contrast with the healthy oral mucosa where no MAGE-A expression profile was determined (Figure 4 and 5). A surgical resection and further histopathological examination was finally performed which revealed an early invasive carcinoma of the oral mucosa (Figure 6).

## Materials and methods

### Isolation of RNA

Total RNA was extracted from cells harvested by cytobrush using the RNA-Bee extraction method according to the manufacturer's protocol (AMS Biotechnology, Europe). In addition, RNA quality and quantity was assessed by the "Lab-ona-Chip" method (Bioanalyzer 2100; Agilent Technologies, Palo Alto, CA) according to the manufacturer's instructions.

The quality of RNA was assessed using the One Step RT-PCR Kit (Qiagen, Hilden, Germany), amplifying cDNA using glyceraldehyde-3-phosphate-dehydrogenase (GAPDH)-specific primers and analyzing the GAPDH-PCR products on a 2% agarose gel. To exclude the false positive results generated by amplifying genomic DNA sequences that were not totally eliminated by DNA digestion, purified RNA from each specimen was tested for amplification of genomic GAPDH using the specific primers for PCR. GAPDH-PCR products were then analyzed on a 2% agarose gel. Only RNA isolations showing the specific amplification product in RT-PCR and no visible band for genomic amplifications were analyzed for the subsequent procedures. All the incisional biopsies and cytologies were investigated by the Institute of Pathology at the University of Erlangen-Nuremberg.

### Reverse transcription and real time PCR

cDNAs from the total RNA were synthesized using the High Capacity cDNA Archive Kit (Cat. 4322171; Applied Biosystems, CA, USA) according to the manufacturer's protocol. Real time RT-PCR analyses were done using QuantiTect Primer Assays (Qiagen; [Hs_QT01841224 QuantiTect Primer Assay] for MAGE-A3 and [Hs_ QT01841225 QuantiTect Primer Assay] for MAGE-A4). For normalization, GAPDH was used [Hs_GAPDH_QT000792471 QuantiTect Primer Assay]. The detection of mRNA was performed with the ABI Prism 7300 Sequence Detection System (Applied Biosystems, Weiterstadt, Germany). The QuantiTect TM SYBR^® ^green PCR kit (Cat. 204143; Qiagen) was used for PCR amplification. Briefly, 50 ng of cDNA was used for each PCR reaction in a total volume of 25 μl. The cycling conditions used for the real time PCR were as follows: initial denaturation/enzyme activation for 15 min at 95°C followed by 40 cycles of denaturation at 94°C for 15 s, annealing at 55°C for 30 s and elongation at 72°C for 34 s. The amplification and presence of a specific product was verified by melting curve analysis after PCR. A Ct value < 33 was recommended as a positive for the expression of the target gene. All the reactions were run in triplet and verified by a second analysis. Results are displayed in Fig. 4. Formation of undesired side products during PCR that contribute to fluorescence was assessed by melting curve analysis after PCR.

## Discussion

Despite the continuous improvements in surgical reconstruction and radiochemotherapy regimens, early detection and diagnosis of OSCC is still the most effective approach for reducing morbidity and mortality.

Clinical inspection of the oral cavity is not sensitive enough in identifying premalignant and malignant lesions [7,10,11]. Until now, tissue harvesting by scalpel biopsy and subsequent histological examination have been the gold standard for diagnosing premalignant and malignant oral diseases. Consequently, adequate clinical intervention should be based on histopathological findings of the lesion. It is evident that incision biopsies taken of suspicious lesions, which have a limited reproducibility within the whole lesion [4], may result in a more or less aggressive surgical and/or radiochemotherapeutic approach. An inter- and intraobserver variability of histological diagnoses was even described by several authors [12,13]. Therefore, identifying further diagnostic tools remains crucial for receiving the most representative analysis of any suspicious lesions.

Oral cytology, as an adjunctive non-invasive sampling technique, is becoming increasingly important in the early diagnosis and monitoring of precursor lesions and oral cancer. Transepithelial 'field mapping biopsies' within widespread lesions are even more essential for cytological evaluation and further investigation [14].

The diagnostic accuracy of the brush biopsy approach can be significantly increased by molecular tumor marker analysis, which reduces false negative diagnosis due to insufficient diagnostic material [7]. Malignant epithelial transformation is considered a process of multiple genetic and epigenetic alterations which plays an important role in tumorigenesis. Cellular changes occur at the molecular level before appearing at the clinical site or in the pathohistological examination, which makes them essential additive tools for diagnosing cell development and transformation [15,16]. Even high grade dysplasia implies an established predictive prognostic marker for malignant transformation, while low and moderate grade dysplasia still have an unknown risk of further malignant transformation [5]. These entities, however, should also be predicted by the combination of histopathological and molecular changes [17] to ensure correct risk assessment. Early identification of premalignant and malignant molecular cellular changes with a result of early intervention may reduce treatment extent, morbidity and mortality.

In this study, we used MAGE-A3 and A4 encoding genes which are expressed in various tumor types while genetically silent in all normal tissues except testis, placenta and fetal tissues [18]. In the current literature, the multiple expression of MAGE-A antigens in OSCC have been described by several authors [19-22]. Our study group has described a high summative expression pattern of the melanoma antigens (MAGE-A1-A6 and -A12) of more than 93% in OSCC [23,24]. Thus, the detection of these markers, which are highly specific to cellular malignancy, makes them potential markers for early diagnosis and prognosis, and even a target for immunotherapy. We used the real time RT-PCR assay, which detects even small amounts of MAGE-RNA [25,26]. In this case report, we further combined these highly prevalent tumor markers with this sensitive detection method using brush biopsy material for the early diagnosis of OSCC.

Therefore, this method presents a novel approach to the early detection of OSCC patients with high risk (e.g., after an initial diagnosis, tobacco and/or alcohol abuse). Additionally, it is an important aid for further diagnosis and treatment of apparently innocuous oral lesions with an unpredictable malignant transformation rate [11]. This method is transferable to all other tumor markers and tumor entities as it represents a promising alternative to various oncological screening settings.

The malignant tissue alteration of the presented suspicious lesion was uncovered by identifying the high prevalent tumor markers in combination with a high sensitive and specific molecular detection analysis using brush biopsy material.

## Conclusion

Sole morphologic interpretations of histopathological alterations of premalignant and malignant lesions and their predictive malignant risk assessment will become insufficient in the future. Objective techniques, especially in conflicting cytopathological findings, will be necessary for further information about the grade and course of epithelial transformation to assure adequate clinical treatment. Therefore, this novel approach may be the first step to increase sensitivity and specificity and even facilitating objectivity in the early diagnosis of just a few malignant cells.

## Competing interests

The authors declare that they have no competing interests.

## Authors' contributions

PM collected tissue samples and drafted the manuscript. NM carried out the RT-PCR. SS carried out the histology and cytology of the samples. FWN participated in its design. EN conceived of the study and participated in the design of the study. JR carried out the molecular marker and performed statistical analysis. All authors read and approved the final manuscript.

## References

[B1] Patton LL Epstein JB Kerr AR Adjunctive techniques for oral cancer examination and lesion diagnosis: a systematic review of the literature J Am Dent Assoc 2008 139 7 896 905 18594075 10.14219/jada.archive.2008.0276

[B2] Bagan JV Scully C Recent advances in Oral Oncology 2007: epidemiology, aetiopathogenesis, diagnosis and prognostication Oral Oncol 2008 44 2 103 8 10.1016/j.oraloncology.2008.01.008 18252251

[B3] Thomson PJ Field change and oral cancer: new evidence for widespread carcinogenesis? Int J Oral Maxillofac Surg 2002 31 3 262 6 10.1054/ijom.2002.0220 12190131

[B4] Holmstrup P Vedtofte P Reibel J Stoltze K Oral premalignant lesions: is a biopsy reliable? J Oral Pathol Med 2007 36 5 262 6 17448135 10.1111/j.1600-0714.2007.00513.x

[B5] Holmstrup P Vedtofte P Reibel J Stoltze K Long-term treatment outcome of oral premalignant lesions Oral Oncol 2006 42 5 461 74 10.1016/j.oraloncology.2005.08.011 16316774

[B6] Lingen MW Kalmar JR Karrison T Speight PM Critical evaluation of diagnostic aids for the detection of oral cancer Oral Oncol 2008 44 1 10 22 10.1016/j.oraloncology.2007.06.011 17825602 PMC2424250

[B7] Mehrotra R Hullmann M Smeets R Reichert TE Driemel O Oral cytology revisited J Oral Pathol Med 2009 38 2 161 6 19213102 10.1111/j.1600-0714.2008.00709.x

[B8] Poate TW Buchanan JA Hodgson TA Speight PM Barrett AW Moles DR Scully C Porter SR An audit of the efficacy of the oral brush biopsy technique in a specialist Oral Medicine unit Oral Oncol 2004 40 8 829 34 10.1016/j.oraloncology.2004.02.005 15288839

[B9] Kaugars GE Silverman S Jr Ray AK Page DG Abbey LM Burns JC Svirsky JA The use of exfoliative cytology for the early diagnosis of oral cancers: is there a role for it in education and private practice? J Cancer Educ 1998 13 2 85 9 9659626 10.1080/08858199809528522

[B10] Sciubba J Biopsy an essential diagnostic tool. Interview by Phillip Bonner Dent Today 1998 17 7 83 5 9796463

[B11] Sciubba JJ Improving detection of precancerous and cancerous oral lesions. Computer-assisted analysis of the oral brush biopsy. U.S. Collaborative OralCDx Study Group J Am Dent Assoc 1999 130 10 1445 57 10570588 10.14219/jada.archive.1999.0055

[B12] Abbey LM Kaugars GE Gunsolley JC Burns JC Page DG Svirsky JA Eisenberg E Krutchkoff DJ Cushing M Intraexaminer and interexaminer reliability in the diagnosis of oral epithelial dysplasia Oral Surg Oral Med Oral Pathol Oral Radiol Endod 1995 80 2 188 91 10.1016/S1079-2104(05)80201-X 7552884

[B13] Kujan O Khattab A Oliver RJ Roberts SA Thakker N Sloan P Why oral histopathology suffers inter-observer variability on grading oral epithelial dysplasia: an attempt to understand the sources of variation Oral Oncol 2007 43 3 224 31 10.1016/j.oraloncology.2006.03.009 16931119

[B14] Thomson PJ Hamadah O Cancerisation within the oral cavity: the use of 'field mapping biopsies' in clinical management Oral Oncol 2007 43 1 20 6 10.1016/j.oraloncology.2005.12.019 16757199

[B15] Hunter KD Parkinson EK Harrison PR Profiling early head and neck cancer Nat Rev Cancer 2005 5 2 127 35 10.1038/nrc1549 15685196

[B16] Scully C Field JK Tanzawa H Genetic aberrations in oral or head and neck squamous cell carcinoma 2: chromosomal aberrations Oral Oncol 2000 36 4 311 27 10.1016/S1368-8375(00)00021-X 10899669

[B17] Scheifele C Schmidt-Westhausen AM Dietrich T Reichart PA The sensitivity and specificity of the OralCDx technique: evaluation of 103 cases Oral Oncol 2004 40 8 824 8 10.1016/j.oraloncology.2004.02.004 15288838

[B18] Jungbluth AA Busam KJ Kolb D Iversen K Coplan K Chen YT Spagnoli GC Old LJ Expression of MAGE-antigens in normal tissues and cancer Int J Cancer 2000 85 4 460 5 10.1002/(SICI)1097-0215(20000215)85:4<460::AID-IJC3>3.0.CO;2-N 10699915

[B19] Muller-Richter UD Dowejko A Peters S Rauthe S Reuther T Gattenlohner S Reichert TE Driemel O Kubler AC MAGE-A antigens in patients with primary oral squamous cell carcinoma Clin Oral Investig 2009 19488795 10.1007/s00784-009-0292-2

[B20] Kienstra MA Neel HB Strome SE Roche P Identification of NY-ESO-1, MAGE-1, and MAGE-3 in head and neck squamous cell carcinoma Head Neck 2003 25 6 457 63 10.1002/hed.10223 12784237

[B21] Figueiredo DL Mamede RC Proto-Siqueira R Neder L Silva WA Jr Zago MA Expression of cancer testis antigens in head and neck squamous cell carcinomas Head Neck 2006 28 7 614 9 10.1002/hed.20380 16475205

[B22] Lee KD Lee HH Joo HB Lee HS Yu TH Chang HK Jeon CH Park JW Expression of MAGE A 1-6 mRNA in sputa of head and neck cancer patients--a preliminary report Anticancer Res 2006 26 2B 1513 8 16619566

[B23] Ries J Vairaktaris E Mollaoglu N Wiltfang J Neukam FW Nkenke E Expression of melanoma-associated antigens in oral squamous cell carcinoma J Oral Pathol Med 2008 37 2 88 93 18197853 10.1111/j.1600-0714.2007.00600.x

[B24] Ries J Mollaoglu N Toyoshima T Vairaktaris E Neukam FW Ponader S Nkenke E A novel multiple-marker method for the early diagnosis of oral squamous cell carcinoma Dis Markers 2009 27 2 75 84 19893202 10.3233/DMA-2009-0652 PMC3834668

[B25] Schwartz JL Panda S Beam C Bach LE Adami GR RNA from brush oral cytology to measure squamous cell carcinoma gene expression J Oral Pathol Med 2008 37 2 70 7 18197850 10.1111/j.1600-0714.2007.00596.x

[B26] Mecklenburg I Weckermann D Zippelius A Schoberth A Petersen S Prang N Riethmuller G Kufer P A multimarker real-time RT-PCR for MAGE-A gene expression allows sensitive detection and quantification of the minimal systemic tumor load in patients with localized cancer J Immunol Methods 2007 323 2 180 93 10.1016/j.jim.2007.04.006 17540401

